# Detection of *Mycoplasma anatis*, *M*. *anseris*, *M*. *cloacale* and *Mycoplasma* sp. 1220 in waterfowl using species-specific PCR assays

**DOI:** 10.1371/journal.pone.0219071

**Published:** 2019-07-11

**Authors:** Dénes Grózner, Kinga Mária Sulyok, Zsuzsa Kreizinger, Zsuzsanna Rónai, Szilárd Jánosi, Ibolya Turcsányi, Henrik Fülöp Károlyi, Áron Botond Kovács, Márton József Kiss, Dmitriy Volokhov, Miklós Gyuranecz

**Affiliations:** 1 Institute for Veterinary Medical Research, Centre for Agricultural Research, Hungarian Academy of Sciences, Budapest, Hungary; 2 Veterinary Diagnostic Directorate, National Food Chain Safety Office, Budapest, Hungary; 3 Department of Microbiology and Infectious Diseases, University of Veterinary Medicine, Budapest, Hungary; 4 TOLL 96 Kft., Hajdúsámson, Hungary; 5 Center for Biologics Evaluation and Research, U. S. Food and Drug Administration, Silver Spring, Maryland, United States of America; University of Helsinki, FINLAND

## Abstract

*Mycoplasma anatis*, *M*. *anseris*, *M*. *cloacale* and *M*. sp. 1220 colonise geese and ducks, and could be associated with infections of avian respiratory and nervous systems, cause mild to severe inflammation of cloaca and genital tracts, and embryo lethality. Co-occurrence of these *Mycoplasma* species in waterfowl is frequently detected and the identification of these mycoplasmas to the species level at a regular microbiology laboratory is difficult due to their similar morphological, cultural and biochemical properties. Moreover, species differentiation is only possible based on the sequence analysis of the product of a genus-specific PCR assay. Therefore, the aim of the current study was to develop an effective and robust method for the identification of these species in avian clinical specimens. Polymerase chain reaction (PCR) assays using species-specific primers, which target housekeeping genes in order to identify these species, were designed in the present study. The developed PCR assays can precisely identify these four mycoplasmas to the species level directly from DNA samples extracted from clinical specimens, and no cross-amplification was observed among these species and with other well-known avian mycoplasmas. The average sensitivity of the assays was 10^1^−10^2^ genomic equivalents per reaction. These conventional PCR assays can be run simultaneously at the same PCR cycling program, and the species can be differentiated directly (without sequence analysis) by gel electrophoresis due to the specific sizes of the amplicons. In conclusion, the presented species-specific assays were found to be suitable for routine use at regular veterinary diagnostic laboratories and promote the rapid, simple and cost-effective differentiation of these waterfowl *Mycoplasma* species.

## Introduction

*Mycoplasma anatis*, *M*. *anseris*, *M*. *cloacale* and *M*. sp. 1220 are well-known waterfowl mycoplasmas. *M*. *anatis*, *M*. *anseris*, and *M*. *cloacale* are well-characterized, validly published species [1–3], while *M*. sp. 1220 has been isolated in 1983 in Hungary from a goose with clinical inflammation of the phallus [4] but not validly published yet as a novel species. However, biochemical, cultural, morphological and serological properties of *M*. sp. 1220 were comprehensively studied [4,5], which allowed for considering this microorganism as an independent *Mycoplasma* species, with a proposed name *M*. *anserisalpingitis* [6–8]. All these mycoplasmas could be a part of the normal microflora of geese and ducks, which do not demonstrate any clinical signs of mycoplasma infection (i.e., inapparent infections or carriers); however, these *Mycoplasma* species are also associated with some pathological conditions in waterfowl. Clinically manifested mycoplasmosis caused by these *Mycoplasma* species can occur in waterfowl during excessive stress, such as inadequate housing, crowding or extensive egg production [9–11], or due to inappropriate hygienic conditions and inadequate access of waterfowl to water. *M*. *anatis* could cause infection associated with neurological symptoms in ducks under stress conditions [12]. Airsacculitis, peritonitis and increased embryo lethality were also described in experimental infection studies in ducks infected with *M*. *anatis* [13]. However, *M*. *anatis* was also isolated from healthy geese [14]. *M*. *anseris* was associated with airsacculitis, peritonitis and embryo lethality in geese [11] and might have a role in the cloaca and phallus inflammation of ganders [7]. *M*. *cloacale* was associated with egg infertility in geese [10] but could be also isolated from healthy ducks, geese and other bird species [1,4,14,15]. Infections of geese with *M*. sp. 1220 cause the most significant production and economical losses in the goose farming business, especially in Central and Eastern European countries where the goose production is particularly popular [7,8,16,17]. Cloaca and phallus inflammation, salpingitis, airsacculitis, peritonitis and embryo lethality are the most frequent symptoms in the affected geese flocks [10,11,16,18,19]. All these four *Mycoplasma* species are well-known to be mainly vertically transmitted [7,11,20,21]. Co-occurrence (or co-infection) of *M*. *anseris*, *M*. *cloacale* and *M*. sp. 1220 in geese has been frequently observed [7,9]. Co-infections of waterfowl with these mycoplasmas together with other bacterial or viral pathogens may lead to more severe disease manifestations and their consequence [22,23].

The species pairs, *M*. *anatis* and *M*. sp. 1220, and *M*. *anseris* and *M*. *cloacale* have undistinguishable biochemical properties (phosphatase-positive glucose-utilizers and phosphatase-negative arginine-hydrolysers, respectively) and all isolates/strains of these species have typical “fried-egg” colony morphology on solid media. Practically, these species could be identified using serological testing via growth inhibition test (GIT) and immunofluorescent antibody test (IFA); however, these tests are time-consuming, require pure culture of isolates and the qualified reference antisera, which are not available at regular veterinary laboratories in most countries either due to absence of national veterinary mycoplasma reference centres or due to a limited use of animals for production of antibody reagents [4,9,14]. Therefore, the characterisation of the phenotypic features of these mycoplasmas is not sufficient for their routine identification to the species level [4,8]. However, their identification to the species level is essential for deeper understanding of their epidemiology and transmission, and for control and eradication of these infections in commercial waterfowl flocks, as well as for the development of experimental vaccines (e.g. against *M*. sp. 1220 in geese [24]) and assessment of their clinical efficacy.

The identification of these mycoplasmas to the species level is possible with the use of the *Mycoplasma* genus-specific PCR assay, which targets the 16S-23S rRNA intergenic transcribed spacer region (ITS) [25]; however, in this case the species identification is based on direct sequencing of obtained PCR amplicons that is not always feasible at regular veterinary laboratories, requires additional reagents and skills, and practically impossible for PCR products amplified from birds simultaneously co-infected with different mycoplasmas. To the best of our knowledge, no *M*. *anatis*-, *M*. *anseris*- or *M*. *cloacale*-specific PCR assays have been published, and only one study on the species-specific PCR detection and phylogenetic identification of *M*. sp. 1220 isolates in geese from the Russian Federation and Ukraine has been published [8]. The aim of the present study was to improve the detection of these mycoplasmas to the species level by developing species-specific PCR assays, which could be suitable for the robust detection of these mycoplasmas in clinical specimens obtained from waterfowl species.

## Materials and methods

### Selection of species-specific regions

In order to design species-specific primers for detection of *M*. *anatis*, *M*. *anseris*, *M*. *cloacale* and *M*. sp. 1220, several housekeeping genes were randomly selected from the mycoplasma minimal genome set [26,27], which are presented in the genomes of the examined species. The sequences of the selected genes (listed below) were obtained from the recently published complete genomes of *M*. *anatis*, *M*. *anseris* and *M*. *cloacale* type strains [28]. The same genes of *M*. sp. 1220 type strain (ATCC BAA-2147) were obtained from the whole-genome sequence determined by *de novo* sequencing in our laboratory using the Illumina (Illumina Inc., San Diego, USA) next generation sequencing platform (unpublished data). Also, the *rpoB* gene sequences of *M*. sp. 1220 type strain and *M*. sp. 1220 field isolates were previously determined by our group and can be found under GenBank accession numbers EU596576 and MH003302—MH003317, respectively. The genome annotation for these species was performed using the RAST software [29]. The following 16 genes were selected for the analysis for the species-specific regions: the acetate/propionate family kinase (*ackA*), cytidine deaminase (*cdd*), DNA polymerase III subunit gamma/tau (*dnaX*), DNA-directed RNA polymerase subunit beta (r*poB*), glucose-6-phosphate isomerase (*pgi*), ribulose-phosphate 3-epimerase (*rpe*), ATP-dependent DNA helicase (*pcrA*), and transcriptional regulator (*mraZ*) genes. Based on our genome analysis, these genes are presented in genomes of all these four *Mycoplasma* species. In addition, the genes that were found to be unique only for some studied *Mycoplasma* species were also included in this analysis. Thus, the 1-phosphofructokinase (*pfkB*), beta-phosphoglucomutase (*pgmB*), phosphoenolpyruvate-protein phosphotransferase (*ptsP*) and UTP-glucose-1-phosphate uridylyltransferase (*gtaB*) genes are presented only in the genomes of *M*. *anatis* and *M*. sp. 1220 type strains. The arginine deiminase (*arcA*) and 5'-methylthioadenosine nucleosidase (*mtn*) genes appeared only in the genomes of *M*. *anseris* and *M*. *cloacale* type strains. The thymidylate synthase (*thyA*) and deoxycytidylate deaminase (*comEB*) genes were only found in genomes of *M*. *anatis* and *M*. *anseris* type strains, respectively. Based on the genome analysis, all these genes are presented as a single copy in the genomes of these four mycoplasmas.

### Primer design

The sequences of the selected genes were aligned and analysed using the Geneious software [30]. The genes were manually analysed and the primer pairs were designed according to the following three main acceptance criteria: (i) the gene of interest should have at least two regions (20–30 bp) containing as many species-specific nucleotide substitutions as possible, and these short sequence regions should be suitable for design of species-specific primers, i.e., to have similar melting temperatures, and do not form hairpin, or self- and cross-dimers, (ii) the distance between the primers’ regions should be approx. 500–1000 bp, and (iii) the selected species-specific primers should be able to amplify the target gene from all tested isolates/strains of the same species but not from others. Primer design was performed using the NetPrimer software (http://www.premierbiosoft.com/netprimer). The specificity of the primers was analysed *in silico* using BLAST NT algorithm (https://blast.ncbi.nlm.nih.gov/Blast.cgi), as well as *in vitro* to demonstrate a possibility for any cross-amplification among the tested species and with other *Mycoplasma/Acholeplasma* species that could be found in avian clinical specimens (Table 1).

**Table 1:** *Mycoplasma* and *Acholeplasma* isolates/strains used in this study.

*Mycoplasma/Acholeplasma* species	Bacterial strain ID	Host	Sample source	Year	Country	County/State^a^
*M*. *anatis*	NCTC 10156	duck	respiratory tract	1964	United Kingdom	N/A
*M*. *anatis*	MYCAV 314^b^	goose	cloaca	2011	Hungary	N
*M*. *anatis*	MYCAV 315	duck	lung and air sac	2012	Hungary	BK
*M*. *anatis*	MYCAV 317^c^	goose	peritoneum	2013	Hungary	BK
*M*. *anatis*	MYCAV 318	duck	phallus lymph	2013	Hungary	C
*M*. *anatis*	MYCAV 321	duck	phallus lymph	2014	Hungary	BE
*M*. *anatis*	MYCAV 324	duck	phallus lymph	2014	Hungary	C
*M*. *anatis*	K6193A*	wild duck	N/A	N/A	USA	NY
*M*. *anatis*	K6193C*	wild duck	N/A	N/A	USA	NY
*M*. *anseris*	ATCC 49234	goose	phallus	1984	Hungary	N/A
*M*. *anseris*	MYCAV 92	goose	phallus	2011	Hungary	HB
*M*. *anseris*	MYCAV 96	goose	oviduct	2013	Hungary	HB
*M*. *anseris*	MYCAV 339	goose	cloaca	2017	Hungary	HB
*M*. *anseris*	MYCAV 346	goose	follicule	2013	Hungary	BK
*M*. *anseris*	MYCAV 347	goose	phallus lymph	2013	Hungary	BK
*M*. *anseris*	MYCAV 348	goose	phallus lymph	2014	Hungary	BK
*M*. *anseris*	MYCAV 349	goose	cloaca	2012	Hungary	BK
*M*. *anseris*	MYCAV 350	N/A	N/A	2014	Hungary	BAZ
*M*. *anseris*	MYCAV 451	goose	cloaca	2018	Hungary	N
*M*. *anseris*	MYCAV 454	goose	cloaca	2018	Hungary	C
*M*. *anseris*	MYCAV 490	goose	cloaca	2018	Hungary	N/A
*M*. *anseris*	MYCAV 491	goose	phallus lymph	2018	Hungary	N/A
*M*. *anseris*	MYCAV 492	goose	cloaca	2018	Hungary	C
*M*. *cloacale*	NCTC 10199	turkey	cloaca	1975	United Kingdom	N/A
*M*. *cloacale*	MYCAV 335	goose	cloaca	2017	Hungary	SSB
*M*. *cloacale*	MYCAV 336	goose	sperm	2017	Hungary	BA
*M*. *cloacale*	MYCAV 341	goose	cloaca	2017	Hungary	N
*M*. *cloacale*	MYCAV 345	goose	cloaca	2017	Hungary	C
*M*. *cloacale*	MYCAV 351	goose	cloaca	2012	Hungary	HB
*M*. *cloacale*	MYCAV 352	goose	phallus lymph	2012	Hungary	BK
*M*. *cloacale*	MYCAV 353	chicken	lung and trachea	2012	Hungary	BK
*M*. *cloacale*	MYCAV 354	goose	cloaca	2012	Hungary	BK
*M*. *cloacale*	MYCAV 355	goose	phallus	2013	Hungary	N
*M*. *cloacale*	MYCAV 356	goose	cloaca	2013	Hungary	N
*M*. *cloacale*	MYCAV 357	goose	phallus lymph	2014	Hungary	C
*M*. *cloacale*	MYCAV 358	goose	phallus lymph	2014	Hungary	P
*M*. *cloacale*	MYCAV 359	goose	phallus lymph	2014	Hungary	H
*M*. *cloacale*	MYCAV 360	goose	phallus lymph	2014	Hungary	N
*M*. *cloacale*	MYCAV 361	N/A	N/A	2014	Hungary	BAZ
*M*. *cloacale*	MYCAV 362	N/A	follicule and oviduct	2015	Hungary	N
*M*. *cloacale*	MYCAV 363	N/A	phallus lymph	2015	Hungary	N
*M*. *cloacale*	MYCAV 364	goose	cloaca	2012	Hungary	BK
*M*. sp. 1220	ATCC BAA-2147	goose	phallus lymph	1983	Hungary	N/A
*M*. sp. 1220	MYCAV 47^d^	duck	lung and air sac	2012	Hungary	BK
*M*. sp. 1220	MYCAV 54	goose	follicule	2013	Hungary	C
*M*. sp. 1220	MYCAV 62	goose	phallus and testis	2013	Hungary	BK
*M*. sp. 1220	MYCAV 179	goose	trachea	2015	Hungary	C
*M*. sp. 1220	MYCAV 203	goose	phallus lymph	2015	Hungary	KE
*M*. sp. 1220	MYCAV 221	goose	cloaca	2015	Hungary	C
*M*. sp. 1220	MYCAV 245	goose	phallus lymph	2016	Hungary	C
*M*. sp. 1220	MYCAV 269	goose	follicule	2016	Hungary	P
*M*. sp. 1220	MYCAV 271	goose	phallus lymph	2016	Hungary	C
*M*. sp. 1220	MYCAV 275	goose	sperm	2016	Hungary	P
*M*. sp. 1220	MYCAV 342	goose	trachea	2017	Hungary	N/A
*M*. sp. 1220	MYCAV 343	goose	follicule	2017	Hungary	N/A
*M*. sp. 1220	MYCAV 344	goose	cloaca	2012	Hungary	N/A
*M*. sp. 1220	MYCAV 421	goose	cloaca	2018	Hungary	C
*M*. sp. 1220	MYCAV 494	goose	phallus lymph	2018	Hungary	N/A
*M*. sp. 1220	31848**	goose	oviduct	2003	Hungary	N/A
*M*. sp. 1220	31948**	goose	ovum	2003	Hungary	N/A
*M*. sp. 1220	32328**	goose	testis	2004	Hungary	N/A
*M*. *columbinasale*	ATCC 33549	pigeon	turbinate	1981	United Kingdom	N/A
*M*. *columborale*	ATCC 29258	pigeon	trachea	1978	Japan	N/A
*M*. *gallinaceum*	ATCC 33550	chicken	trachea	1981	United Kingdom	N/A
*M*. *gallinarum*	ATCC 19708	fowl	respiratory tract	1968	United Kingdom	N/A
*M*. *gallisepticum*	ATCC 19610	chicken	respiratory tract	1697	United Kingdom	N/A
*M*. *gallopavonis*	ATCC 33551	turkey	air sac	1981	United Kingdom	N/A
*M*. *iners*	ATCC 19705	chicken	respiratory tract	1977	United Kingdom	N/A
*M*. *iowae*	ATCC 33552	turkey	air sac	1981	United Kingdom	N/A
*M*. *meleagridis*	NCTC 10153	turkey	N/A	1976	United Kingdom	N/A
*M*. *pullorum*	ATCC 33553	chicken	trachea	1981	United Kingdom	N/A
*M*. *synoviae*	NCTC 10124	chicken	hock joint	1969	United Kingdom	N/A
*A*. *laidlawii*	NCTC 10116	N/A	sewage	1967	United Kingdom	N/A

^a^Abbreviations: N/A data not available; BA Baranya; BAZ Borsod-Abaúj-Zemplén; BE Békés; BK Bács-Kiskun; C Csongrád; H Heves; HB Hajdú-Bihar; KE Komárom-Esztergom; N Nógrád; NY New York; P Pest; SSB Szabolcs-Szatmár-Bereg

^b^97.9% amplicon sequence match with the *dnaX* gene of *M*. *anatis* type strain.

^c^97.7% amplicon sequence match with the *dnaX* gene of *M*. *anatis* type strain.

^d^98.6% amplicon sequence match with the *rpoB* gene of *M*. sp. 1220 type strain.

*The strains were originally received from Naola Ferguson-Noel, University of Georgia, Poultry Diagnostic & Research Center, Athens, GA, USA.

**The strains were originally received from László Stipkovits, RT-Europe Research Center Ltd., Budapest, Hungary.

### Development of species-specific PCR assays

To evaluate the PCR assays designed in this study, a total of 57 *Mycoplasma* isolates/strains were used (Table 1). The type strains were obtained from the reference collections (ATCC and NCTC), and field isolates were previously obtained by the authors during routine diagnostic examinations of clinical specimens from domestic geese, ducks and chickens and from wild ducks. In addition, 28 clinical specimens, including cloaca swabs, follicule tissue, trachea tissue, phallus lymph, and sperm, were also examined in this study (Table 2). These samples were collected during routine clinical examinations or at necropsies of geese and ducks. No ethical approval was required for these sample collections. The tested *Mycoplasma* field isolates and the avian clinical specimens were collected mainly in Hungary between 2003 and 2018 (Table 1). DNA was extracted from the isolates/strains and the clinical specimens using the QIAamp DNA Mini Kit (Qiagen Inc., Hilden, Germany) according to the manufacturers’ instructions. The primary screening and identification of the 57 *Mycoplasma* isolates/strains were performed by the genus-specific PCR assay [25] followed by direct sequencing of the produced amplicons on ABI Prism 3100 automated DNA sequencer (Applied Biosystems, Foster City, USA), and by nucleotide sequence analysis in BLAST database. The 28 clinical specimens were also primary screened for the presence of *Mycoplasma* DNA using the genus-specific PCR [25], and then these samples were further investigated using the species-specific PCRs developed in this study.

**Table 2:** *Mycoplasma* spp. detection in the clinical specimens.

Clinical sample No.	Host	Sample source	Year	County of Hungary^a^	*Mycoplasma* genus-specific PCR^b^	*M*. *anatis*	*M*. *anseris*	*M*. *cloacale*	*M*. sp. 1220
1	goose	cloaca	2015	SSB	+^g^	-	+^d^	+	+
2	goose	cloaca	2015	C	++	+^c^	-	+	+
3	goose	cloaca	2016	SSB	++	+^c^	+	+	+
4	goose	follicule	2016	S	-	-	-	-	-
5	goose	sperm	2016	N/A	++	+^c^	-	+	+
6	goose	sperm	2016	N/A	++	-	-	+^e^	+
7	goose	sperm	2016	N/A	++	+^c^	-	+	+
8	goose	phallus lymph	2016	C	-	-	-	-	-
9	goose	trachea	2016	HB	-	-	-	-	-
10	duck	cloaca	2016	HB	-	-	-	-	-
11	duck	phallus lymph	2016	HB	-	-	-	-	-
12	goose	cloaca	2017	SSB	++	-	+	+	+
13	goose	cloaca	2017	SSB	+^h^	-	+	+	+
14	goose	cloaca	2017	N	++	-	-	+	+
15	goose	cloaca	2017	N	++	-	-	+	+
16	goose	cloaca	2017	C	++	-	+	+	+
17	goose	cloaca	2017	N/A	++	-	-	+	+
18	goose	cloaca	2017	N/A	++	-	-	+	+
19	goose	cloaca	2017	N/A	++	-	+	+	+
20	goose	cloaca	2017	HB	+	-	+	-	-
21	goose	cloaca	2017	HB	++	-	+	-	+^f^
22	goose	cloaca	2017	HB	+	-	+	-	-
23	goose	cloaca	2017	HB	++	-	+	-	+
24	goose	sperm	2017	BA	+	-	-	+	-
25	goose	sperm	2017	BA	+^g^	-	+	+	-
26	goose	cloaca	2017	BA	++	-	+	+	+
27	goose	cloaca	2017	BA	+	-	-	+	-
28	goose	phallus lymph	2018	N/A	+^h^	-	+	-	+
Total No. of positive samples					24	4	13	18	18

^a^Abbreviations: N/A data not available; BA Baranya; C Csongrád; HB Hajdú-Bihar; N Nógrád; S Somogy; SSB Szabolcs-Szatmár-Bereg

^b^+ indicates only one, ++ indicates two PCR products

^c^97.8–97.9% amplicon sequence match with the *dnaX* gene of *M*. *anatis* type strain.

^d^100% amplicon sequence match with the *pcrA* gene of *M*. *anseris* type strain.

^e^99.5% amplicon sequence match with the *dnaX* gene of *M*. *cloacale* type strain.

^f^98.5% amplicon sequence match with the *rpoB* gene of *M*. sp. 1220 type strain.

^g^DNA sequence chromatogram indicated mixed infection.

^h^Neither the number of PCR products nor the DNA sequence chromatograms indicated mixed infection.

**Fig 1:**
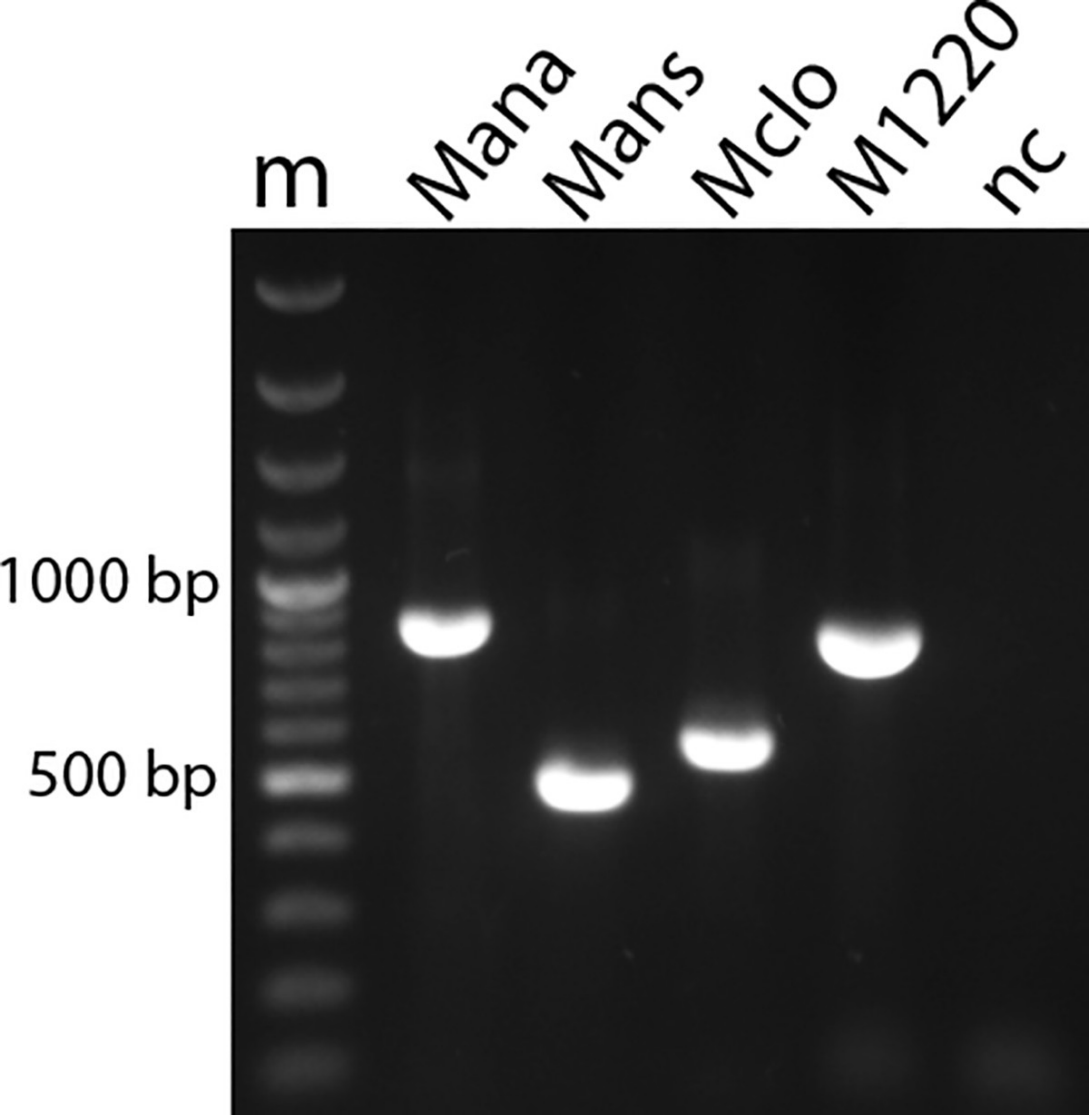
Sizes of the PCR amplicons of *Mycoplasma* species generated by the species-specific PCR assays. Abbreviations: m—molecular weight marker (GeneRuler 100 bp Plus DNA Ladder, Thermo Fisher Scientific, Waltham, USA); Mana—product from the *M*. *anatis*-specific assay; Mans—product from the *M*. *anseris*-specific assay; Mclo—product from the *M*. *cloacale*-specific assay; M1220—product from the *M*. sp. 1220-specific assay; nc—negative control.

The species-specific PCR assays were carried out in 25 μl total volume, containing 2 μl target DNA sample, 5 μl 5X Green GoTaq Flexi Buffer (Promega Inc., Madison, WI), 2 μl MgCl_2_ (25mM), 0.5 μl dNTP (10 mM, Qiagen Inc., Hilden, Germany), 2 μl of each primer (10 pmol/μl) and 0.25 μl GoTaq DNA polymerase (5 U/μl). The final primer set was selected based on specificity and sensitivity criteria detailed in section “Assessment of specificity and sensitivity of the developed assays”. The sequences of the designed primers and the sizes of the species-specific amplicons are provided in Table 3 (Fig 1). The actual sequences of the species-specific amplicons are provided in the S1 File. Thermocycling parameters were 95°C for 5 min, followed by 40 cycles at 95°C for 1 min, 61°C for 1 min and 72°C for 1 min and a final elongation step was performed at 72°C for 5 min. Detection of PCR products with the expected molecular weights were confirmed by electrophoresis in 1% TBE-agarose gel stained with ECO Safe Nucleic Acid Staining Solution (Pacific Image Electronics Co., Ltd, New Taipei City, Taiwan) followed by UV visualization. Ultrapure water was used as a negative control matrix in parallel to monitor for cross-contamination. The amplification quality of extracted mycoplasma DNA was tested using the genus-specific PCR primers [25].

**Table 3:** Primers’ sequences and sizes of amplicons for the designed species-specific PCR assays.

*Mycoplasma* species	Target gene	Primer sequence (5'-3')	Size of amplicon (bp)
*M*. *anatis*	*dnaX*	CAGAGATCAGTCTGTTTTAGAATTACTTT	895
		TTTCTCAGATGCTTGTGAAATACAACTT	
*M*. *anseris*	*pcrA*	CTAAAAACTCCTAAAGACTTAGAAGAATC	504
		ATCCTCACCTTCATCATTTTCTGTATA	
*M*. *cloacale*	*dnaX*	TTCATCCGATAAGTTAAAACCTTGTT	591
		AAAACTGCTTTTGTATTTTTAGAATATAGT	
*M*. sp. 1220	*rpoB*	CCGTGATACTGCTCAATTCGAA	857
		TAGAAGTATAAACATCATCCTTAACAAGCT	

### Assessment of specificity and sensitivity of the developed assays

The specificity of the PCR assays was analysed by DNA testing of the following avian *Mycoplasma* and *Acholeplasma* type strains: *M*. *anatis* (NCTC 10156), *M*. *anseris* (ATCC 49234), *M*. *cloacale* (NCTC 10199), M. *columbinasale* (ATCC 33549), *M*. *columborale* (ATCC 29258), *M*. *gallinaceum* (ATCC 33550), *M*. *gallinarum* (ATCC 19708), *M*. *gallisepticum* (ATCC 19610), *M*. *gallopavonis* (ATCC 33551), *M*. *iners* (ATCC 19705), *M*. *iowae* (ATCC 33552), *M*. *meleagridis* (NCTC 10153), *M*. *pullorum* (ATCC 33553), *M*. sp. 1220 (ATCC BAA-2147), *M*. *synoviae* (NCTC 10124) and *Acholeplasma laidlawii* (NCTC 10116).

In order to test the sensitivity of the PCR assays, all designed primer pairs were tested with the corresponding DNA of the *M*. *anatis* (NCTC 10156), *M*. *anseris* (ATCC 49234), *M*. *cloacale* (NCTC 10199) and *M*. sp. 1220 (ATCC BAA-2147) type strains 10-fold serially diluted (10^6^−10^0^) in nuclease-free water. Template copy numbers (corresponding to genomic equivalents or GE in the current study) per reaction for these strains were calculated based on the used DNA concentrations using an online dsDNA copy number calculator (https://cels.uri.edu/gsc/cndna.html) and the following genome size information: 956,093 bp for *M*. *anatis* (GenBank accession number CP030141), 750,010 bp for *M*. *anseris* (CP030140), 659,552 bp for *M*. *cloacale* (CP030103) and 908,787 bp for *M*. sp. 1220.

During the selection of the final primer set, primers which demonstrated cross-amplification between the tested *Mycoplasma/Acholeplasma* strains were excluded from further study. The sensitivity of the PCR assays using different primer pairs for the detection of a certain waterfowl *Mycoplasma* species were compared visually during gel electrophoresis. Primer pairs showing the highest sensitivity and specificity were chosen for the final study.

In order to evaluate the performance of the designed assays in mixed infections, the DNA of the waterfowl *Mycoplasma* type strains (containing 10^6^ GE) were mixed in a ratio of 1:1:1:1 and tested. Also, DNA mixes were created with one DNA sample containing 10^3^ GE and the other three are represented with 10^6^ GE in a ratio of 1:1:1:1, and submitted to the corresponding waterfowl *Mycoplasma*-specific assay (i.e. DNA mix of 10^3^ GE *M*. *anatis*, 10^6^ GE *M*. anseris, 10^6^ GE *M*. *cloacale* and 10^6^ GE *M*. sp. 1220 in the *M*. *anatis*-specific PCR assay).

### Confirmation of the assays’ specificity by PCR amplicon sequencing

In order to demonstrate that the obtained PCR products were species-specifically amplified we performed direct DNA sequencing of all amplicons from the type strains and amplicons generated from some selected isolates/strains and clinical specimens. All PCR products amplified from goose specimens using the *M*. *anatis*-specific PCR assay (i.e., MYCAV 314 and 317 isolates and clinical specimens No. 2, 3, 5, 7) were also sequenced to confirm their species identity because the presence of *M*. *anatis* in geese is not commonly reported. Randomly selected amplicons were also sequenced from the *M*. *anseris-* and *M*. *cloacale-*specific PCRs to verify their species identity (clinical specimens No. 1 and 6, respectively). In the case of the *M*. sp. 1220-specific PCR, product of the MYCAV 47 isolate originating from a duck, and a randomly selected amplicon (clinical specimen No. 21) were sequenced to confirm their species identity. The *Mycoplasma* genus-specific PCR products of clinical specimens No. 1, 13, 25 and 28, which according to the results of the developed species-specific PCR assays represented mixed mycoplasma DNA samples due to natural mycoplasma co-infections in geese, were also sequenced in order to analyse their DNA sequence chromatograms for the presence of mixed sequences. All analysed amplicons were purified using the QIAquick PCR Purification Kit (Qiagen Inc., Hilden, Germany) and directly sequenced with the same primers used for their PCR amplification. The sequences obtained from the type strains, isolates/strains and clinical specimens are deposited in GenBank under accession numbers MK532897 –MK532910.

## Results

Sixteen mycoplasma housekeeping genes were analysed in the study, out of which eight genes were found suitable for the design of species-specific primers based on the above-mentioned acceptance criteria. Therefore, the species-specific primers were designed for the following genes: the *dnaX*, *pgmB*, *ptsP* and *thyA* genes to detect *M*. *anatis*; the *dnaX*, *pcrA* and *mtn* genes to detect *M*. *anseris*; the *dnaX* and *mtn* genes to detect *M*. *cloacale*; and the *rpoB*, *pfkB* and *pgmB* genes to detect *M*. sp. 1220. These primary PCR assays based on these designed primers showed species-specificity *in silico*; however, *in vitro* analyses revealed cross-amplification among the species in one case (between *M*. *anatis* and *M*. *anseris*, primer sequences 5’ TCAAATTCAAAAATTGTTCCTTGC 3’ and 5’ ATGTGTTCTAATTGAAGCCATTTTAAT 3’ specific for *dnaX* gene), which was excluded from further examinations. The rest of the omitted primers showed one- to two-fold lower sensitivity in the species-specific PCR amplification based on UV visualization, hence, they were excluded from further analyses. According to the combined results on the primers specificity and sensitivity performed by testing the DNA samples of the mycoplasma type strains, PCR assays, which did not demonstrate any cross-amplification with other tested *Mycoplasma/Acholeplasma* strains and showed superior sensitivity, were selected as the final assays. The *dnaX* gene-based PCR assays were the most suitable for precise species identification of *M*. *anatis* and *M*. *cloacale* (sensitivity of these assays was 10^2^ GE per reaction), the *pcrA* gene-based PCR assay was accepted for species identification of *M*. *anseris* (sensitivity of 10^1^ GE per reaction), and the *rpoB* gene-based PCR assay was suitable for precise identification of *M*. sp. 1220 (sensitivity of 10^2^ GE per reaction). As end-point PCR systems were designed in the study, the yield of a PCR product does not provide quantity result after UV visualization. However, the visual detection of the PCR products in the sensitivity tests means that the minimum GEs detectable in the samples are 10^1^ in the case of *M*. *anseris*-specific PCR and 10^2^ in the cases of *M*. *anatis*-, *M*. *cloacale*- and *M*. sp. 1220-specific assays. The similarity between the *dnaX* gene of *M*. *anatis* and *M*. *cloacale* is 51.32% and the highest similarity among the bonding sites of the *M*. *anatis*- and *M*. *cloacale*-specific primer pairs is 54.55%; therefore, cross-amplification among the two species in the specific PCR assays is unlikely. Species identification was successful in all cases during the tests of the *M*. *anatis*, *M*. *anseris*, *M*. *cloacale* and *M*. sp. 1220 type strain DNA mixes, the presence of the other waterfowl pathogen mycoplasmas did not have any effect on the assays’ performance.

The result of the species-specific PCRs obtained with the testing of 28 clinical specimens is provided in Table 2. Among the 28 clinical specimens 5 were negative in all PCR assays, 4 specimens were positive for one *Mycoplasma* species, 9 specimens for two species, other 9 specimens for three species and one specimen was positive for all tested *Mycoplasma* species. Sequences of the amplicons obtained from these species-specific assays on the clinical specimens and isolates/strains showed their 97.7–100% identity with the corresponding sequences of the type strains (Tables 2 and 3, and the S1 File), confirming that the assays were able to amplify the gene regions specific to the given species.

The *Mycoplasma* genus-specific PCR [25] amplified PCR products of approx. 460 bp from *M*. *anatis* and *M*. sp. 1220, and smaller products of approx. 370 bp from *M*. *anseris* and *M*. *cloacale*. This assay revealed two amplicons at the corresponding molecular weights when *M*. *anatis / M*. sp. 1220 and *M*. *anseris* / *M*. *cloacale* co-occurred in the samples, with the exception of three samples. In these three cases (No. 1, 13 and 28) a single amplicon was amplified by the genus-specific PCR assay, while the species-specific PCR assays were able to detect mixed infections of *M*. sp. 1220, along with *M*. *anseris* and/or *M*. *cloacale* in these geese (Table 2). Moreover, both *M*. *anseris* and *M*. *cloacale* were detected in a clinical specimen (No. 25) by the developed assays, which showed the same amplicon size by the genus-specific PCR assay. The DNA sequence chromatogram analysis performed on the genus-specific PCR amplicons from the clinical samples No. 1 and 25 revealed major sequences of *M*. *sp*. 1220 and *M*. *anseris*, respectively, with evidence of well-visible secondary peaks indicating a mixed DNA sequence due to the natural co-infections. However, the DNA sequence chromatogram analysis performed on the genus-specific PCR amplicons from the clinical samples No. 13 and 28 also revealed major sequences of *M*. *sp*. 1220 and *M*. *anseris*, respectively, but did not demonstrate any detectable mixed sequences (S2 File). This discrepancy between the results of the *Mycoplasma* genus-specific PCR and the developed species-specific PCR assays could be associated with a substantial difference of these assays in term of their detection specificity and/or sensitivity for different *Mycoplasma* species, especially if the analysed mycoplasma sample presents a mixed DNA sample (see discussion below).

## Discussion

*M*. *anatis*, *M*. *anseris*, *M*. *cloacale* and *M*. sp. 1220 can cause infections in waterfowl clinically manifested with respiratory and neurological symptoms, inflammation of cloaca and genital tracts, and embryo lethality, which eventually result in significant economic and poultry production losses. Co-infection of waterfowl with these *Mycoplasma* species that share similar phenotypic characteristics is frequent, and therefore the isolation of pure culture and the precise identification of these species by biochemical and serological tests are challenging issues in routine veterinary laboratories. The species-specific PCR assays developed in the present study enable the species-specific identification of these waterfowl *Mycoplasma* species and expedite laboratory diagnosis of these infections (including mixed infections), providing valuable support for the selection and the use of adequate control and/or feasible treatment of affected birds [31,32].

The nucleotide sequences of the 16S rRNA genes of *M*. *anatis* and *M*. sp. 1220 demonstrate significant similarity (90.5% according to Sprygin et al., 99.1% based on our observation) and therefore cannot be used for the unambiguous differentiation between these two species [8]. Also, the high percentage of similarity of the 16S rRNA genes and the 16S-23S rRNA ITS sequences between *M*. *anseris* and *M*. *cloacale* (98.3% and 87.3%, respectively) [33] makes these genetic markers less suitable for precise identification of these species. The previously published PCR assays targeting these genetic regions for the detection of mycoplasmas [25,34–36] demonstrated low reliability in the identification of waterfowl *Mycoplasma* species. Moreover, these methods almost always require the direct sequencing of amplicons for the species confirmation, which can be difficult or impossible in cases of co-infections with different *Mycoplasma* species, as well as in laboratories in which DNA sequencing technologies are not available.

The *Mycoplasma* genus-specific PCR assay [25] provides different amplicon sizes for certain *Mycoplasma* species (e.g. 90bp difference between amplicon sizes of *M*. *anatis / M*. sp. 1220 and *M*. *anseris / M*. *cloacale*) due to the length variation of the 16S-23S rRNA ITS among the species [33]. Therefore, amplicons at different molecule weights already indicate co-occurrence of mycoplasma species. However, the detection of only one amplicon does not necessarily mean the presence of only one species, and in certain cases even the analysis of the chromatograms could not indicate the presence of co-occurring species. In the current study, comparison of the results of the primary screening of clinical samples using the genus-specific PCR assay with the results of the developed species-specific PCR assays revealed discrepancies in certain cases. The observed differences could be the consequences in the ratio between the mixed DNAs presented in the clinical samples, which can influence their equally efficient detectability [37]. The presented results confirm that the 16S-23S ITS-based PCR assay is well suitable for primary detection or screening of clinical specimens for the presence of mycoplasma DNA; however, it is unsuitable for the detection and identification of all presented species in samples from mixed mycoplasma infection cases, which could be common. This observation highlights the importance of the utilisation of other genetic targets to avoid potential false identifications and incorrect diagnostic results for mycoplasma detection in clinical specimens.

*M*. sp. 1220 represents a serious threat to the geese industry, especially in Central and Eastern Europe, therefore the detection of this agent is required for a comprehensive laboratory diagnostic analysis of goose clinical specimens. Previous attempts have been made to design primers for the detection and specific identification of this species based on the sequences of the *rpoB*, DNA polymerase III subunit alpha (*dnaE*), elongation factor (*fusA*), and the pyruvate kinase (*pyk*) genes [8]; however, those primers were not analysed for the detection sensitivity of *M*. sp. 1220 in avian clinical samples. The primers for the *M*. sp. 1220-specific PCR assay described in this study also target the *rpoB* gene but the sensitivity of the assay was established to ensure its applicability for the diagnostic use.

The PCR assays developed in this study were able to identify these *Mycoplasma* species in new or rarely-observed waterfowl hosts. According to the available literature *M*. sp. 1220 has been previously isolated only from geese [7]; however, in this study this species was also identified in a domestic duck using the *Mycoplasma* genus-specific PCR [25] and this result was confirmed by the newly designed species-specific PCR assay, as well as, by the DNA sequence analysis of the PCR amplicons. Fibrin deposition on the visceral serous membranes of the duck in this case was also consistent with the pathological evidences observed in *M*. sp. 1220-infected geese [19]. To our best knowledge this is the first official isolation of *M*. sp. 1220 (isolate MYCAV 47) from an avian species other than goose. The partial *rpoB* gene (1997 bp long) was also sequenced for this *M*. sp. 1220 isolate (MYCAV 47; GenBank accession number MH003302) using primers and conditions described previously [33], and the nucleotide sequencing analysis demonstrated its 99% identity to the *rpoB* gene of the type strain of *M*. sp. 1220.

*M*. *anatis* normally colonises ducks and it was rarely detected in geese [7,14]. In the present study, eight *M*. *anatis* isolates were identified in the tested specimens out of which two isolates originated from geese (MYCAV 314 and 317). The observed pathological changes such as the fibrinous peritonitis and lymphohistiocytic infiltration in the lungs of these geese were similar to the pathological evidences reported for *M*. *anatis-*infected ducks [12]. Moreover, 15% (n = 4/26) of the examined clinical specimens from geese were found to be positive for *M*. *anatis* according to our results generated using the newly designed species-specific PCR assays. DNA sequence analysis of the *dnaX* amplicons from the two isolates and the four clinical specimens revealed their 97.7–97.9% sequence identity to the same gene of *M*. *anatis* type strain (S1 File). This high level of nucleotide identity is sufficient for the identification to the species level and demonstrates the adequate detection specificity of the *M*. *anatis*-based PCR assay. In addition, the partial *rpoB* gene (1994 bp long) was also sequenced for these two *M*. *anatis* isolates originated from geese (MYCAV 314 and 317; GenBank accession numbers MH003311 and MH003313, respectively) using primers and conditions as mentioned above, and the corresponding sequencing result demonstrated their 99% identity to the *rpoB* gene of the type strain of *M*. *anatis*. Thus, the species identity of *M*. sp. 1220 isolate from a duck and *M*. *anatis* isolates from geese was clearly demonstrated in both cases.

The developed assays clearly discriminated the targeted waterfowl pathogen *Mycoplasma* species in the examined samples, isolates and type strains; however, as the majority of the samples originated from a restricted geographic region, further examinations on a wider selection of samples would enable the determination of the robustness of the assays.

Finally, the species-specific PCR assays developed in the current study showed high sensitivity and specificity, enabling rapid, precise and reliable identification of these four *Mycoplasma* species in waterfowl specimens, and therefore proved to be suitable and cost-effective method for the identification of these mycoplasmas in routine veterinary laboratory diagnostics. In addition, these assays can be run simultaneously using the same thermal cycling PCR conditions on conventional PCR equipment.

## Supporting information

S1 File Comparison of nucleotide sequences of the amplicons generated for the type strains, field isolates and mycoplasma amplicons detected in clinical specimens using the designed species-specific PCR assays. Primers (grey) and single nucleotide polymorphisms (black) are highlighted in the sequences.
(PDF)

S2 File Comparison of DNA sequence chromatograms from samples with natural co-infections generated by the *Mycoplasma* genus-specific PCR assay. (PDF)
