# Supplementary figures and images for: Detection of Mycoplasma anatis, M. anseris, M. cloacale and Mycoplasma sp. 1220 in waterfowl using species-specific PCR assays
Source: PLoS One. 2019 Jul 11;14(7):e0219071. doi: 10.1371/journal.pone.0219071 (PMC6622482; doi:10.1371/journal.pone.0219071)

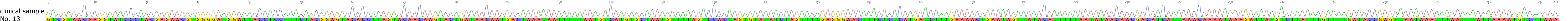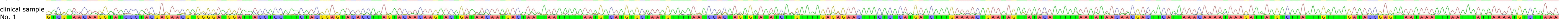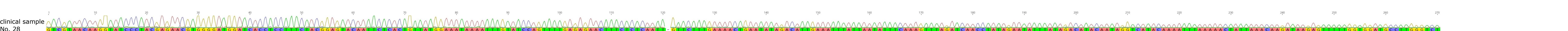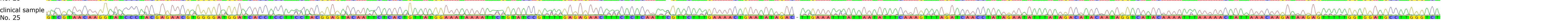

Supplement: S2 File — (PDF) [file pone.0219071.s002.pdf]
