# Supplementary material for: Detection of Mycoplasma anatis, M. anseris, M. cloacale and Mycoplasma sp. 1220 in waterfowl using species-specific PCR assays
Source: PLoS One. 2019 Jul 11;14(7):e0219071. doi: 10.1371/journal.pone.0219071 (PMC6622482; doi:10.1371/journal.pone.0219071)
Supplement: S1 File — Primers (grey) and single nucleotide polymorphisms (black) are highlighted in the sequences. (PDF) [file pone.0219071.s001.pdf]

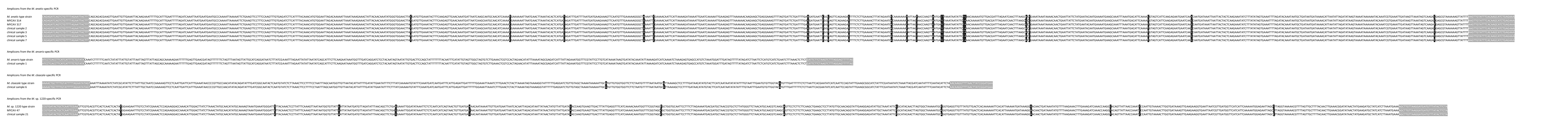
Amplicons from the M. anatis-specific PCR
M. anatis type strain
MYCAV 314
MYCAV 317
clinical sample 2
clinical sample 3
clinical sample 5
clinical sample 7
Amplicons from the M. anseris-specific PCR
M. anseris type strain
clinical sample 1
Amplicons from the M. cloacale-specific PCR
M. cloacale type strain
clinical sample 6
Amplicons from the M. sp. 1220-specific PCR
M. sp. 1220 type strain
MYCAV 47
clinical sample 21
